# Cold Physiology: Postprandial Blood Flow Dynamics and Metabolism in the Antarctic Fish *Pagothenia borchgrevinki*

**DOI:** 10.1371/journal.pone.0033487

**Published:** 2012-03-13

**Authors:** Erik Sandblom, William Davison, Michael Axelsson

**Affiliations:** 1 Department of Biological & Environmental Sciences, University of Gothenburg, Gothenburg, Sweden; 2 School of Biological Sciences, University of Canterbury, Christchurch, New Zealand; Max-Delbrück Center for Molecular Medicine (MDC), Germany

## Abstract

Previous studies on metabolic responses to feeding (i.e. the specific dynamic action, SDA) in Antarctic fishes living at temperatures below zero have reported long-lasting increases and small peak responses. We therefore hypothesized that the postprandial hyperemia also would be limited in the Antarctic fish *Pagothenia borchgrevinki.* The proportion of cardiac output directed to the splanchnic circulation in unfed fish was 18%, which is similar to temperate fish species. Contrary to our prediction, however, gastrointestinal blood flow had increased by 88% at twenty four hours after feeding due to a significant increase in cardiac output and a significant decrease in gastrointestinal vascular resistance. While gastric evacuation time appeared to be longer than in comparable temperate species, digestion had clearly commenced twenty four hours after feeding as judged by a reduction in mass of the administered feed. Even so, oxygen consumption did not increase suggesting an unusually slowly developing SDA. Adrenaline and angiotensin II was injected into unfed fish to investigate neuro-humoral control mechanisms of gastrointestinal blood flow. Both agonists increased gastrointestinal vascular resistance and arterial blood pressure, while systemic vascular resistance was largely unaffected. The hypertension was mainly due to increased cardiac output revealing that the heart and the gastrointestinal vasculature, but not the somatic vasculature, are important targets for these agonists. It is suggested that the apparently reduced SDA in *P. borchgrevinki* is due to a depressant effect of the low temperature on protein assimilation processes occurring outside of the gastrointestinal tract, while the gastrointestinal blood flow responses to feeding and vasoactive substances resemble those previously observed in temperate species.

## Introduction

The Antarctic ocean comprises the coldest and most thermally stable marine environments in the world with water temperatures constantly remaining below 0°C in many places [1], [2], [3]. Temperature has a direct effect on the metabolism of ectotherms, and the metabolic rate (i.e. oxygen consumption; MO_2_) in Antarctic fishes is low compared with temperate species (see [4]). While available information for Antarctic species is limited, the increase in metabolism following feeding (i.e. the specific dynamic action, SDA) is also suggested to be unusually long-lasting and with a reduced peak response due to the low temperature (see [5], [6], [7]). Reports on SDA duration in Antarctic fish range from ∼9 days in *Notothenia neglecta* [8] to ∼16 days in *Harpagifer antarcticus* [9]. This is considerably longer than in comparable temperate species (see [5]). Similarly, gastric evacuation times (GET) are typically longer in polar fishes [10], [11], but general conclusions must be made with caution given the limited number of studies and large differences in experimental protocols.

In temperate fishes, between 10 to 40% of cardiac output (Q) is distributed to the gastrointestinal tract in the unfed state [12], [13]. Depending on e.g. meal size and temperature, gastrointestinal blood flow (q_gast_) can increase nearly two-fold after a meal (i.e. postprandial hyperemia). This is mainly thought to serve convection of absorbed nutrients, and possibly to support a postprandial increase in gastrointestinal tissue metabolism (see [12]). Even for temperate species, however, the relationships between postprandial hyperaemia, SDA and gastric evacuation time are far from completely understood (see [12]); and nothing is known about gastrointestinal blood flow distribution in polar fish. Given the apparently slow SDA response in Antarctic fishes it could be hypothesised that any postprandial hyperaemic response should be limited, but currently no information is available to substantiate this idea.

The neuro-humoral control of gastrointestinal blood flow is complex. It involves both the autonomic and the enteric nervous system, as well as hormones and local factors [12]. In temperate species, gastrointestinal vascular resistance (R_gast_) increases and reduces q_gast_ during metabolic challenges such as exercise and environmental hypoxia [14] to prioritize blood flow to more immediately important organs. Several observations suggest that adrenergic mechanisms are important for this redistribution. Catecholamines reduce q_gast_ *in vivo* [15], and pharmacological or surgical inhibition of adrenergic nerve transmission reduce gastrointestinal vascular resistance and abolish reductions in q_gast_ during exercise and hypoxia [14], [16]. Whether the control of the gastrointestinal vasculature in Antarctic fish differs from temperate species is unknown, but several studies suggest that vasomotor control in Antarctic fish may differ from temperate species [17]. For example, the humoral catecholaminergic stress response is very limited [18], [19]. Even so, exogenous administration of adrenaline and the vasoactive hormone angiotensin II in *Pagothenia borchgrevinki* and *Trematomus bernacchii* increase dorsal aortic blood pressure (P_da_) by increasing stroke volume (SV) and cardiac output (Q), while systemic vascular resistance (R_sys_) is largely unchanged [20], [21], [22]. This contrasts with the profound increases in R_sys_, with only limited changes in Q, which are typically observed in temperate fish (see e.g. [23]).

The overall objective of the present study was to characterize gastrointestinal blood flow dynamics in *P. borchgrevinki* using surgically implanted blood flow probes *in vivo* during digestion and following administration of vasoactive drugs. First, the cardiorespiratory response to a standardized meal was investigated. By measuring whole-animal oxygen consumption along with q_gast_ we tested the hypotheses that the postprandial metabolic response and the gastrointestinal hyperaemia are reduced in this species. Second, gastric evacuation times were determined for instrumented and uninstrumented fish to test the previous notion that gastric evacuation is unusually extended in Antarctic fishes. Third, given the unusual neuro-humoral cardiovascular control in these fish, and the complete lack of knowledge regarding the control of the gastrointestinal vasculature, the hemodynamic responses to injections of adrenaline and angiotensin II were explored.

## Materials and Methods

### Animal collection and holding

Experiments were conducted at Scott Base, Antarctica (77°51′S, 166°45′E) in October and November. *Pagothenia borchgrevinki* used for *in vivo* cardiorespiratory experiments had a body mass range of 97–136 g (mean: 121±4 g). Fish used in gastric evacuation experiments had a body mass range of 58–129 g (mean: 88±4 g). All fish were caught fishing from the ice of McMurdo Sound and transported to Scott Base. Fish were kept in tanks with through-flowing McMurdo Sound seawater at a temperature of −0.5 to +0.5°C. The photoperiod was 24 h daylight during this period and the fish were not fed. Individuals with signs of X-cell gill disease were discarded (see e.g. [24], [25]). All animal husbandry conditions and experimental protocols reported in this paper were approved by the University of Canterbury animal ethics committee (approval number 2007/41R).

### Surgical procedures

Individual fish were anaesthetised in seawater containing MS-222 (100 mg l^−1^; Sigma, St Louis, MO, USA). Prior to surgery, the stomach of the fish was emptied of any remaining food by flushing the stomach with seawater using a large syringe. The fish was then weighed and transferred to an operating cradle where the gills were continuously irrigated with seawater at 0–3°C containing a lower dose of the anaesthetic (50 mg l^−1^). To measure cardiac output (Q), the ventral aorta was dissected free close to the base of the fourth gill arch and a Transonic 1.5SL transit-time blood flow probe (Transonic Systems Inc, Ithaca, New York, USA) was positioned around the vessel without damaging the pericardium. To measure gastrointestinal blood flow, the celiacomesenteric artery was accessed via an incision on the right side of the fish. The vessel was placed in a Transonic V-type transit-time blood flow probe. The position of the probe was above the bifurcation of the celiac and the mesenteric arteries, which meant that total blood flow to the gastrointestinal tract was recorded [12]. Temperature effects on the probe readings were compensated for. The incision was closed with interrupted 3-0 or 4-0 silk sutures. Due to a limited number of flow probes we could not instrument all fish with probes for both cardiac output and gastrointestinal blood flow. Consequently, in some fish only gastrointestinal blood flow, and not cardiac output, was measured. To measure dorsal aortic blood pressure (P_da_), the dorsal aorta was cannulated via the third efferent branchial artery with a PE-50 catheter tipped with ∼1 cm of PE-10 and filled with heparinised (100 IU ml^−1^) saline (1% NaCl) [20]. The PE-10 tip section was spliced to the main catheter using a ∼1 cm section of flexible polyurethane tubing as described previously [26]. The flow probe leads and the catheter were attached to the skin using 3-0 or 4-0 silk suture. After surgery the fish was placed in either a respirometer (see below for details) or in a plastic holding tube that were covered to minimise visual disturbance of the fish. The fish were left to recover from surgery for ∼24 h.

### Experimental setup and data acquisition

In approximately half of the instrumented fish, oxygen consumption (MO_2_) was determined using a Perspex respirometer. It was placed in a larger outer tank supplied with a continuous flow of aerated seawater at ∼0°C. A submersible pump circulated the water in the respirometer and an oxygen-meter (Oxi340i/SET, WTW, Weilheim, Germany) placed in-line with the mixing pump was used to record oxygen levels. A second pump, controlled by a custom-built digital time relay, was used to flush and close the respirometer at regular intervals. MO_2_ was calculated from the drop in oxygen levels in the closed state. Oxygen levels never declined below 85% saturation and background oxygen consumption was found to be negligible.

The dorsal aortic catheter and the two flow probe leads were externalized via a small opening at the top of the respirometer. P_da_ was recorded by connecting the catheter to a DPT-6100 pressure transducer (pvb Medizintechnik, Kirchseeon, Germany) that was regularly calibrated against a static column of seawater. The signal from the transducer was amplified using a 4Champ amplifier (Somedic, Hörby, Sweden). The flow probe leads were connected to a Transonic blood flow meter (Model T206, Transonic Systems Inc, Ithaca, New York, USA). Voltage signals from the recording equipment were fed into a PowerLab system (ADInstruments Pty Ltd, Castle Hill, Australia) connected to a laptop computer running LabChart Pro software (ADInstruments Pty Ltd, Castle Hill, Australia) for data acquisition and subsequent data analysis.

### Experimental protocol: Neurohumoral control of gut blood flow

After the post-surgical recovery period, the hemodynamic effects of adrenaline (5 nmol kg^−1^) and angiotensin II [(salmon; Asn^1^, Val^5^) 0.1 nmol kg^−1^] were first tested. These dosages were chosen based on previous studies in *P. borchgrevinki* [20], [21]. The drugs were administered via the dorsal aortic catheter as 1 ml kg^−1^ bolus injections followed by 0.2 ml of saline to clear the catheter dead space. The cardiovascular effects of these doses wore off within less than one hour.

### Experimental protocol: Cardiorespiratory responses to feeding

Approximately twenty four hours after the initial pharmacological experiments, the effects of forced feeding on cardiovascular function were assessed [15], [27], [28]. Baseline cardiorespiratory variables were first recorded in unfed fish for several hours. MS-222 was then added to the water and when the fish had just lost its righting reflex it was quickly force-fed with pieces of fish white muscle amounting to 2% of body mass using blunt forceps. This ration is close to the average food content of 1.6%, but lower than the maximum found food content of 5.5%, found in freshly caught *P. borchgrevinki* [29]. A ration of 2% of body mass was therefore considered to represent a physiologically relevant stimulus in this species. Cardiorespiratory variables were again recorded after 24 h to estimate the post-prandial cardiorespiratory response. After the experiment, gastric evacuation time (GET) for the instrumented fish was determined as described below for uninstrumented fish.

### Experimental protocol: Gastric evacuation

The gastric evacuation time was determined in a separate group of non-instrumented *P. borchgrevinki* [28]. Individual fish were anesthetized in MS-222 and any remaining food items in the stomach were removed by flushing the stomach with seawater. Individual fish were then marked with a colored thread sutured dorsally for identification. Fish were held in a common tank receiving through-flowing seawater and left to recover from the handling procedures for several days. At the start of the experiment, individual fish were again anesthetized and force-fed with 2% of body mass as described for instrumented fish. No signs of feed regurgitation were observed following this procedure. Six randomly selected fish were then terminally sampled after 24, 48 and 72 h and remaining food contents in the stomach were collected and the wet weight was determined to estimate GET [28].

### Calculations and statistical analysis

Heart rate (*f*_H_) was determined from pulsatile flow and pressure traces. Stroke volume (*V*_S_) and systemic vascular resistance (R_sys_) were calculated as *V*_S_ = Q/*f*_H_ and R_sys_ = P_da_/Q, respectively. Gastrointestinal vascular resistance (R_gast_) was calculated as R_gast_ = P_da_/q_gast_. The above calculations assumed that central venous blood pressure remained close to zero [21]. Oxygen consumption (MO_2_) was calculated as described previously [30].

Paired comparisons of cardiorespiratory variables in unfed and post-prandial conditions were performed using a non-parametric Wilcoxon signed-ranks test and differences in gastric evacuation time were analyzed using two-tailed t-tests. The dynamic effects of injected pharmacological agents were analyzed with one-way repeated-measures ANOVA followed by Dunnet's post-hoc test to identify values that were significantly different from baseline. Reported values are means (+S.E.M) unless otherwise stated. The fiduciary limit was 5% for all statistical comparisons.

## Results

### Baseline cardiorespiratory status and the effects of forced feeding

**Figure 1:**
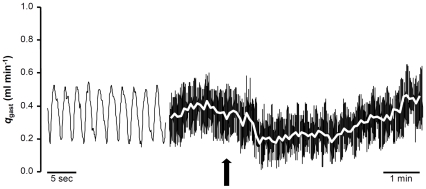
Representative gastrointestinal blood flow trace. The trace was recorded from a 120 g *P. borchgrevinki* at ∼24 hours postprandial. Vertical arrow indicates when the experimenter taps on the respirometer provoking a precipitous reduction in blood flow that subsequently returns to baseline.

Despite the relatively small size of *P. borchgrevinki*, recordings of hemodynamic variables including q_gast_ were of satisfying quality. Figure 1 shows a representative original gastrointestinal blood flow trace and the marked transient reduction in gastrointestinal blood flow occurring as the experimenter gently tapped on the holding tank.

**Figure 2:**
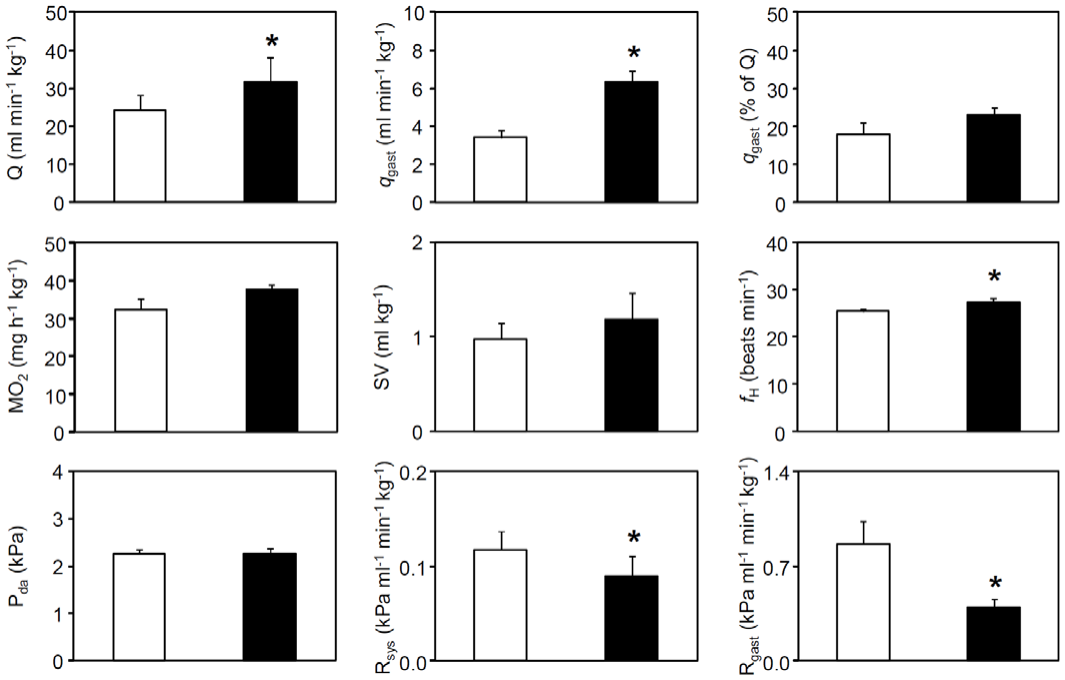
Cardiorespiratory status before and after feeding. *P. borchgrevinki* were force fed with fish white muscle amounting to ∼2% of body mass. Open bars are unfed fish (N = 9–14) and closed bars are 24 h after feeding (N = 5–8). The variables are cardiac output (Q), gastrointestinal blood flow (q_gast_), oxygen consumption (MO_2_), stroke volume (SV), heart rate (*f*_H_), dorsal aortic blood pressure (P_da_), systemic vascular resistance (R_sys_) and gastrointestinal vascular resistance (R_gast_). Values are means (+S.E.M.) and * denotes significantly different value (p<0.05) from the unfed control.

Cardiorespiratory values in unfed fish and 24 h after force-feeding with a meal amounting to 2% of body mass are summarized in Figure 2. Baseline MO_2_ was 32.3±2.9 mg h^−1^ kg^−1^ and did not change significantly after feeding, while q_gast_ increased significantly from a baseline value of 3.4±0.4 to 6.4±0.6 ml min^−1^ kg^−1^ at 24 hours postprandial. The increase in gastrointestinal blood flow was due to a significant increase in Q from 24.4±4.0 to 31.7±6.5 ml min^−1^ kg^−1^, in combination with a significantly reduced resistance of the gastrointestinal vasculature from 0.86±0.17 to 0.39±0.06 kPa ml^−1^ min^−1^ kg^−1^. In those fish where we were able to measure Q and q_gast_ simultaneously, the calculated percentage of Q allocated to the gastrointestinal circulation did not change significantly after feeding (17.9±3.1% of Q in unfed vs. 22.9±2.1% of Q after feeding). The increase in Q was mediated by a significantly increased *f*_H_ from 25.3±0.7 to 27.2±0.9 beats min^−1^. Baseline P_da_ was 2.3±0.1 kPa and remained unchanged after feeding despite the increased Q because R_sys_ decreased significantly from 0.12±0.02 to 0.09±0.02 kPa ml^−1^ min^−1^ kg^−1^. Continuous measurements in several fish from the time of feeding strongly suggested that oxygen consumption had clearly not peaked before the 24 h recording (data not shown).

### Gastric evacuation time

**Figure 3:**
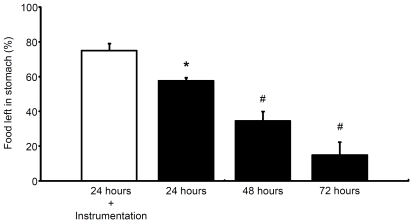
Gastric evacuation time. Values were taken at 24, 48 and 72 hours following forced feeding of *P. borchgrevinki* with fish white muscle amounting to ∼2% of body mass. The open bar represents the instrumented fish used in cardiorespiratory *in vivo* experiments (N = 9) and closed bars represent non-instrumented fish (N = 6–7). Values are means (+S.E.M.). * denotes significantly different value (p<0.05) between instrumented and uninstrumented fish at 24 hours postprandial and # denotes significantly different value (p<0.05) from the preceding sampling time in uninstrumented fish.

GET in uninstrumented and surgically instrumented *P. borchgrevinki* following forced feeding with a meal amounting to ∼2% of body mass is presented in figure 3. For uninstrumented fish, the amount of food left in the stomach was 58±2%, 34±6% and 15±8% at 24, 48 and 72 hours postprandial, respectively. This decrease in stomach content was statistically significant at each sampling time. At 72 h post-feeding, three out of seven fish had no food left in the stomach. Surgical instrumentation clearly delayed digestion because in the instrumented fish 75±4% of the administered food remained in the stomach at 24 hours after feeding. This was significantly greater than the 58±2% at 24 hours in the uninstrumented fish.

### Gastrointestinal blood flow responses to adrenaline and angiotensin II

**Figure 4:**
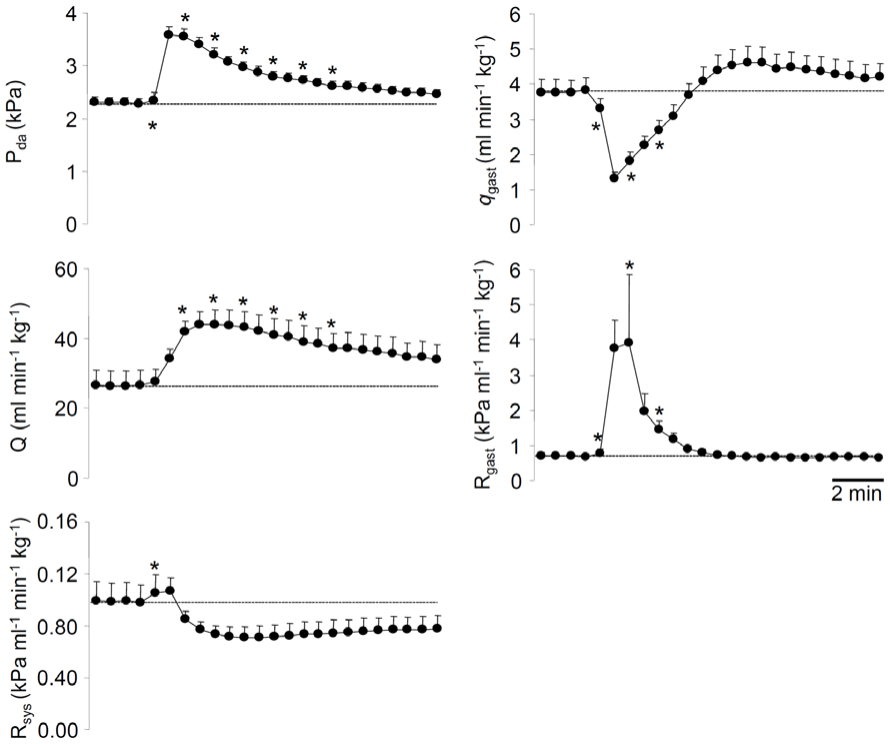
Cardiovascular responses to adrenaline. Adrenaline (5 nmol kg^−1^) was injected into unfed *P. borchgrevinki* (N = 7–12). The variables are dorsal aortic blood pressure (P_da_), cardiac output (Q), systemic vascular resistance (R_sys_), gastrointestinal blood flow (q_gast_) and gastrointestinal vascular resistance (R_gast_). Dotted vertical lines extrapolate baseline values for visual aid. Values are means (+S.E.M.) and * denotes significantly different value (p<0.05) from the pre-injection control period.

The effects of intra-arterially administered adrenaline (5 nmol kg^−1^) on selected cardiovascular variables are illustrated in figure 4. While the adrenaline injection increased cardiac output by 66% within 2 minutes, *q*_gast_ decreased significantly by 188% within less than 1 minute. This reduction in gastrointestinal blood flow was mediated by a 451% increase in *R*_gast_, which meant that the proportion of Q being directed to the gastrointestinal tract decreased transiently to around 5%. The increase in Q was due to a significant increase in stroke volume (data not shown), while heart rate did not change in any consistent way. Dorsal aortic blood pressure increased rapidly following the injection and peaked within 1 minute. This moderate hypertension was primarily mediated by the increase in Q, because R_sys_ only displayed a modest ∼10% increase during the first minute after the injection, and then, if anything, dropped below baseline levels (data not shown).

**Figure 5:**
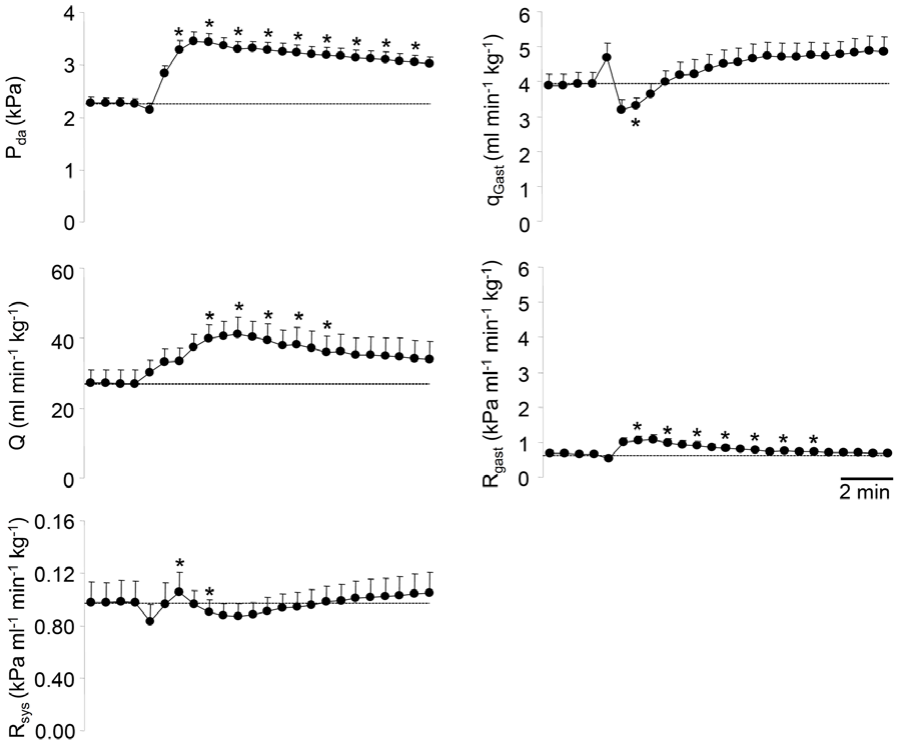
Cardiovascular responses to angiotensin II. Angiotensin II (5 nmol kg^−1^) was injected into unfed *P. borchgrevinki* (N = 7–12). The variables are dorsal aortic blood pressure (P_da_), cardiac output (Q), systemic vascular resistance (R_sys_), gastrointestinal blood flow (*q*_gast_) and gastrointestinal vascular resistance (R_gast_). Dotted vertical lines extrapolate baseline values for visual aid. Values are means (+S.E.M.) and * denotes significantly different value (p<0.05) from the pre-injection control period.

The effects of angiotensin II [(salmon; Asn^1^, Val^5^) 0.1 nmol kg^−1^] on selected cardiovascular variables are presented in figure 5. Similar to adrenaline, angiotensin II reduced blood flow to the gastrointestinal tract by 31% during the first minute due to a 67% increase in *R*_gast_. Furthermore, Q increased by up to 52% due to an increased SV, while heart rate decreased (data not shown). Compared with the response to adrenaline, however, the increase in cardiac output in response to angiotensin II developed slower and peaked after 3–4 minutes. P_da_ increased significantly following the injection, which again was largely due to increased Q because R_sys_ did not change in any consistent way that could explain the hypertension.

## Discussion

This is the first report of gastrointestinal blood flow dynamics in an Antarctic fish. Despite the rather invasive surgical procedures required to implant the gastrointestinal blood flow probe; routine values for heart rate, blood pressure and cardiac output reported here are similar to previous studies in *P. borchgrevinki* involving instrumentation with branchial catheters and a ventral aortic flow probe [20], [21], [26], [31], [32]. This suggests that the surgery required to implanting the gastrointestinal blood flow probe did not result in markedly greater post-surgical stress. In fact, routine MO_2_ in previous studies on uninstrumented *P. borchgrevinki* bracket the value of 32 mg h^−1^ kg^−1^ reported here for instrumented fish [4].

### Routine gastrointestinal blood flow

In absolute terms, routine q_gast_ in unfed *P. borchgrevinki* at ∼0°C (3.4 ml min^−1^ kg^−1^) is low compared with previous measurements in temperate fish species. For example, total blood flow to the gastrointestinal tract (i.e. the sum of coeliac and mesenteric artery blood flow) is 9.0 ml min^−1^ kg^−1^ in Red Irish lord (*Hemilepidotus hemilepidotus*) at 7–9°C [15]; 7.6 ml min^−1^ kg^−1^ in Atlantic cod (*Gadus morhua*) at 10–11°C [14]; 12–18 ml min^−1^ kg^−1^ in Chinook salmon (*Oncorhynchus tshawytscha*) at 8–11°C [33] and 9.6 and 13.8 ml min^−1^ kg^−1^ in sea bass (*Dicentrarchus labrax*) at 16 and 23°C, respectively [28], [34]. Nevertheless, the calculated percentage of Q directed to the gut in unfed fish in the present study was 18%, which is within the 10–40% range previously reported for a collection of temperate teleost species [12].

While it is clear from the present study that a relatively large proportion of cardiac output is directed to the gastrointestinal tract even in this Antarctic fish that has a low basal metabolism, it is not immediately obvious why such a large proportion of the total blood flow is allocated to the gastrointestinal tract. To the best of our knowledge, available information on the gastrointestinal metabolic demands in fishes is limited to one study in the gulf toadfish (*Obsanus beta*) where the oxygen consumption of the gastrointestinal tract was estimated to account for only ∼5% of the total oxygen consumption in both the unfed state and during SDA using isolated intestinal tissue [35]. Thus, while it appears from a metabolic point of view that this tissue is over-perfused, the high blood flow must have other functions beyond those of house-keeping gas and nutrient convective purposes. For example, in seawater fish it might be crucial to maintain a relatively high and continuous blood flow through the splanchnic circulation for osmoregulatory purposes (see [33]). Furthermore, the liver is perfused by the hepatic portal vein that drains the gastrointestinal vasculature [36]. Given the central role of this organ in energy metabolism and various detoxification processes, this may be another explanation as to why the splanchnic circulation receives such a large proportion of the total blood flow.

### Postprandial blood flow responses and relations to oxygen consumption

Twenty four hours after forced feeding gastrointestinal blood flow had increased by 88%. There was no clear postprandial redistribution of blood flow in *P. borchgrevinki* because overall Q increased significantly and the relative proportion of Q directed to the gastrointestinal tract did not change (Fig. 2). This hemodynamic pattern is consistent with results from temperate fish species [14], [15], [37], but different from mammals where cardiac output does not normally increase following a meal and the increase in gastrointestinal blood flow is mainly due to redistribution of blood flow away from other vascular beds (see [12]).

The SDA is generally considered to represent the sum of the metabolically demanding processes involved in the ingestion, digestion, absorption and assimilation of a meal [5], [6]. In that respect it was interesting to notice that at twenty four hours after feeding q_gast_ had nearly doubled, and digestive processes in the stomach had clearly already commenced as judged from the reduction in the mass of the feed (Fig. 3, see discussion below), but oxygen consumption was still unchanged. This finding is important because it indicates that the contribution of digestive processes in the stomach to SDA is small and suggests that the postprandial increase in q_gast_ does not primarily have a metabolic role, but may rather be related to convection of the absorbed nutrients or some other function. This line of reasoning is also consistent with the idea that the SDA is mainly attributed to metabolic processes occurring outside of the gastrointestinal tract, with the assimilation of amino acids into proteins representing the main cost [6], [38], [39]. Thus, the lack of an increase in metabolism at twenty four hours postprandial observed here in *P. borchgrevinki* suggests that those processes may be markedly slowed in Antarctic fishes living at temperatures around zero. This is also in line with a negative correlation between body temperature and SDA duration in temperate fish species [6] and an unusually slowly developing SDA response remaining for up to 16 days in Antarctic fish [9]. An obvious limitation of the present study is that we were not able to record gastrointestinal blood flow from individual fish for the full duration of the postprandial period. We are therefore unable to tell whether q_gast_ had reached its peak within the twenty four hour recording period, and for how long blood flow to the gastrointestinal tract remained elevated after a meal. Future studies combining long-term recordings of gastrointestinal blood flow dynamics and postprandial oxygen consumption in Antarctic fishes with a slow SDA could prove fruitful to get a better temporal resolution and thereby understanding of the dynamics and relationships between gastrointestinal perfusion and SDA in vertebrates.

### Gastric evacuation time

We are only aware of one previous study reporting on GET in *P. borchgrevinki*. Montgomery and co-workers [29] used serial slaughter of freshly caught specimens to fit an exponential curve to the decay in stomach contents. The half-life (i.e. when 50% of the initial amount is cleared from the stomach) was calculated for various mollusks and crustaceans found in the natural diet. These values ranged between 8 and 34 hours, with crustaceans possessing hard exoskeletons taking longer to clear. When the half-life is calculated in the same way from the present experiments, where *P. borchgrevinki* were force fed fish white muscle, a value of 32 hours is obtained. While this value is at the high end of the ranges calculated by Montgomery et al. [29], it can probably be explained by the differences in experimental protocols. The previous study did not take a potential lag-phase of digestion into account and instead assumed that digestion starts immediately as food is ingested. It is also possible that stress associated with anesthesia and handling during the force feeding procedure in the present study delayed digestion [28], [40].

While GET typically decreases with increasing temperature in fish [6], [41], comparisons of gastric evacuation rates among species from different thermal environments is complicated by differences in e.g. feeding habits and experimental protocols. When the 34% food left in the stomach at forty eight hours postprandial in the present study is used as a reference and compared with previous studies on temperate species fed fish white muscle, it is suggested that GET is relatively slow in *P. borchgrevinki*. For example, sea bass (*Dicentrarchus labrax*) at 16°C force fed with a similar amount of food as in the present study (2.9% of body mass) had only 7% remaining in the stomach after the same time [28]. Shorthorn sculpin (*Myoxocephalus scorpius*) spontaneously feeding at 10°C, had a similar amount of food (35%) remaining in the stomach at forty eight hours postprandial, but these fish consumed much larger meals amounting to 8–10% of body mass [27]. In the Arctic cod (*Boreogadus saida*) at temperatures similar to the present study (−1.4–0.5°C), half-lives ranged between 36 and 160 hours [10], [11]. However, differences in experimental protocols make inter-specific comparisons and general conclusions problematic and more research is clearly required to get a better picture of the effects of sub-zero temperatures on the digestive processes in fish.

### Neurohumoral control of gastrointestinal blood flow

Consistent with studies in temperate fish species, adrenaline increased gastrointestinal vascular resistance significantly [14], [15], [16], [42]. The systemic vascular resistance was largely unaffected by this agonist, which is consistent with previous studies in Antarctic fish [20], [21], [22]. Thus, these findings suggest that there is a profound difference in the importance of adrenergic vascular control mechanisms between the systemic and the gastrointestinal vasculature in *P. borchgrevinki*. Several observations indicate that it is primarily neural catecholamines that affect the splanchnic vasculature in this species. First, a recent study on *P. borchgrevinki* indicated substantial innervation from catecholaminergic nerves of blood vessels in the gastrointestinal tract as revealed with immunohistochemistry, while very few catecholaminergic neurons were found adjacent to vessels in the skeletal musculature [31]. Adrenergic nerve blockade with bretylium caused a 23% reduction in R_sys_ *in vivo*, a response that was speculated to be primarily due to relaxation of the gastrointestinal vasculature [31]. However, since q_gast_ was not recorded, and the systemic vascular resistance is the sum of the resistances of the somatic and the gastrointestinal vascular beds, it was not possible to conclusively determine whether this was the case. Second, routine and post-stress levels of circulating catecholamines are normally very low in Antarctic fishes [18], [19], [43], and so a major importance of these hormones for gastrointestinal vascular control seems unlikely. Third, the rapid startle-induced reduction in gastrointestinal blood flow in response to tapping on the holding tank in the present study indicates a neural mechanism (Fig. 1). Whether this response can be entirely explained by increased adrenergic nervous discharge will await confirmation by future studies using e.g. adrenergic nerve blockers.

As far as we are aware this is the first study examining the effects of angiotensin II on gastrointestinal blood flow dynamics in any species of fish. The increase in gastrointestinal vascular resistance to this agonist is consistent with a functional renin-angiotensin system in teleosts [44], including Antarctic species [45]. Nevertheless, the mode of action of angiotensin II is complicated and from the initial studies reported here it cannot be determined whether the vasoactive influence on the gastrointestinal vasculature was due to interactions with adrenergic control systems as found in temperate fishes [46], [47], [48]. Nevertheless, the increase in dorsal aortic blood pressure resulting from increased cardiac output following adrenaline and angiotensin II is consistent with previous studies [20], [21], [22]. Collectively, these observations suggest that the heart and the gastrointestinal vasculature, but not the somatic vasculature, are important targets for adrenaline and angiotensin II in *P. borchgrevinki*.
